# Guided Endodontics—Potential and Limitations

**DOI:** 10.1111/adj.70012

**Published:** 2025-10-26

**Authors:** Thomas Connert, Elias Walter, Leander Benz, Falk Schwendicke, Wadim Leontiev

**Affiliations:** ^1^ Department of Periodontology, Endodontology and Cariology University Center for Dental Medicine Basel UZB, University of Basel Basel Switzerland; ^2^ Department of Conservative Dentistry and Periodontology University Hospital, LMU Munich Munich Germany

## Abstract

Guided Endodontics has emerged as a digital treatment concept designed to overcome the challenges associated with conventional access cavity preparation in teeth with pulp canal calcification. Calcification of the pulp (also referred to as obliteration or mineralization), often caused by trauma or chronic irritative stimuli, presents a substantial clinical obstacle during endodontic treatment. The advent of computer‐assisted technologies such as static and dynamic navigation systems has enabled highly precise and minimally invasive localization of root canal orifices, even in cases of severe calcification. This narrative review explores the clinical indications, technical workflows, and current evidence for both static and dynamic Guided Endodontics, including their limitations and future directions. Numerous in vitro and clinical studies have shown that guided access preparation results in higher precision, reduced dentine loss, and increased success rates compared to freehand techniques, even when performed by less experienced clinicians. While static navigation provides excellent accuracy for straight canals, its application in posterior teeth and curved canals remains limited. Dynamic navigation, in contrast, offers greater intraoperative flexibility but requires significant training and costly equipment. Future developments, including augmented reality integration and MRI‐based workflows, may further expand the applicability of Guided Endodontics. However, the current techniques are limited by cost, planning time, and the necessity for advanced imaging. Despite these challenges, Guided Endodontics has the potential to transform the management of calcified canals and represents a significant step forward in minimally invasive endodontics.

**Summary**

- Guided endodontics enables clinicians to perform highly accurate, minimally invasive access cavity preparations in teeth with pulp canal calcification—situations that often challenge even experienced endodontists.
- This technology improves canal detection rates and reduces the risk of iatrogenic damage, thereby increasing the success of root canal treatment and preserving natural dentition.

## 1 Introduction

Calcification of the coronal pulp tissue complicates access to the root canal orifices during root canal treatment. Calcifications may extend to the root canal orifice, mid‐canal, or even apical regions, thereby increasing the complexity of root canal preparation. Dental trauma, especially dislocation injuries, represents a major etiological factor for root canal calcification. In addition, non‐traumatic factors may also play a significant role in its development. Pulpal calcification is not pathological per se; however, subsequent pulpal pathosis may necessitate root canal treatment in affected teeth. During conventional access cavity preparation, complications and technical errors may arise depending on the extent of calcification, potentially compromising the success of root canal treatment and thus endangering tooth preservation. Through preoperative three‐dimensional imaging and the ability to digitally plan the access cavity and transfer planning to the clinical situation by using a template, endodontic access cavities can be prepared precisely and minimally invasively, even in cases of severe calcification extending to the apical third. First introduced and subsequently termed Guided Endodontics, this technique has been widely studied and is now well integrated into clinical endodontic practice.

## 2 Endodontic Access Cavity Preparation

The preparation of an adequate endodontic access cavity represents the first invasive step in endodontic therapy. The objective is to locate the root canal orifice(s) and to establish a pathway for root canal preparation instruments to the root canal(s) [1]. A straight‐line access facilitates further instrumentation of the root canal using either hand files or rotary nickel‐titanium instruments, thereby reducing the risk of instrument fractures or preparation errors like ledge formation or canal transportation [2]. Consequently, the access cavity plays a crucial role in the overall success of root canal therapy. Access preparation is typically performed using rotary instruments such as diamond‐coated burs or carbide round burs [3], and the access cavity should be dimensioned conservatively to preserve as much tooth structure as possible for biomechanical stability [4]. However, it is important to note that an inadequately sized access cavity can increase the risk of missed canals; for example, a second canal in mandibular incisors [5]. In addition, coronal tooth discoloration may occur, for example, in maxillary anterior teeth of young patients with large root canal lumens if tissue remnants or root canal filling material; for example, sealer, remain in the pulp horns due to insufficient access cavity dimensions.

Endodontic access cavity preparation can be complicated by a variety of factors. While anatomical variations, such as dens invaginatus, are relatively rare [6], more commonly encountered challenges arise from extensive coronal restorations, including crowns or bridges, which can hinder the visualization of the root canal orifice. One contributing factor may be an altered angulation of the coronal tooth axis [7], which typically serves as a key reference point for the clinician.

In addition, calcifications of the pulp chamber and root canals may significantly impede access. Calcification of the root canal system leads to narrowing of the canals and a shift in the position of the canal orifice from a coronal to a more apical location [8].

## 3 Pulp Canal Calcification

Pulp canal calcifications can arise as a delayed consequence of dental trauma. They are particularly common following milder types of luxation injuries, with calcification of the pulpal tissue observed in approximately 15%–40% of cases over time [9, 10]. For calcified tissue to be deposited, the neurovascular bundle at the apical foramen must remain intact, given that this process relies on the activity of vital pulp cells. In contrast, more severe injuries such as intrusion, avulsion, or significant lateral luxation often result in complete disruption of the apical neurovascular bundle, particularly in teeth with a smaller diameter of the apical foramen, making spontaneous pulp revascularization during healing unlikely and increasing the likelihood of pulpal necrosis.

Beyond dental trauma, several other etiological factors for pulp canal calcification have been identified in the literature. These include deep carious lesions [11], restorative procedures [12], vital pulp therapy [13], and orthodontic treatment [14]. In elderly individuals, mild yet chronic mechanical or thermal stimuli have also been associated with progressive calcification of the root canal system [15].

At the molecular level, the biological mechanisms underlying the deposition of calcified tissue in the pulp have been extensively studied and are now better understood. A key trigger appears to be pulp tissue hypoxia, which may result from compression of the apical neurovascular bundle due to occlusal trauma, luxation injuries, or aggressive orthodontic tooth movement. Similarly, inflammatory processes can lead to localized swelling within the pulp, impairing vascular perfusion to adjacent areas. The release of the neuropeptide substance P [16] has been identified as an important mediator in the cascade that leads to hard tissue formation, most commonly referred to in the literature as calcification. However, because this process involves not only calcium but also other mineral deposits, the term mineralization is equally appropriate. The expression pulp canal obliteration refers to the same phenomenon and was predominantly used in earlier publications.

Pulp canal calcification is typically asymptomatic and often detected incidentally on radiographic examination or suggested clinically by a yellowish discoloration of the affected tooth. Although these teeth frequently exhibit a negative response to pulp sensibility testing, the deposition of calcified tissue within the pulp–dentine complex is mediated by vital hard tissue‐forming cells. As such, this process should not be regarded as inherently pathological but rather as a reactive adaptation of the pulp in response to various irritative or traumatic stimuli.

Historically, early root canal treatment was recommended upon radiographic detection of pulp canal calcification to address two key clinical challenges [17]. First, as the canal orifice becomes progressively displaced apically, the risk of perforation during access cavity preparation increases. Second, a total loss of coronal access to the root canal orifice may ultimately necessitate endodontic surgery.

Currently, there is general consensus that root canal treatment is not indicated unless clinical and radiographic signs of pulpal or periapical pathology are present [18, 19], since calcification alone does not constitute a pathological condition.

However, findings from clinical research in the field of dental traumatology state that pulp necrosis may develop as a delayed consequence in teeth with canal calcifications in a certain proportion of cases. The likelihood of progression to pulp necrosis increases over time and has been reported in the literature to occur in up to 27% of cases [20]. This transition may be preceded by a symptomatic or asymptomatic phase of pulpitis. Once necrosis of the pulp tissue occurs, bacterial invasion is likely even in cases of “radiologically completely calcified root canals”. In these cases, with calcifications extending to the apex with no visible canal lumen, histological studies on extracted teeth have clearly demonstrated a residual, though very narrow, canal lumen containing pulp tissue is usually still present [21]. Microorganisms may infiltrate the root canal system through lateral canals or enamel‐dentine cracks, gaining access to dentinal tubules and the main root canal space. In a necrotic state, the pulp is no longer capable of immune defense, resulting in the development of infected pulp necrosis. The presence of microorganisms and their toxins at the apical foramen induces a localized immune response. This immune activation triggers a cascade of inflammatory mediators, ultimately leading to increased osteoclastic activity and resulting in inflammation‐driven bone resorption at the apex of the affected tooth, manifesting as apical periodontitis. The occurrence of pulp necrosis with apical periodontitis constitutes a clear indication for root canal treatment.

Providing adequate root canal treatment in cases with advanced calcifications was described to be technically demanding with a heightened risk of complications due to difficulties in access cavity preparation and canal instrumentation. A retrospective study from 1982 evaluated the incidence of procedural errors and the 4‐year treatment outcomes in incisors presenting with post‐traumatic pulp canal calcification and periapical pathology [22]. Root canal treatments were performed on 54 teeth with root canal calcifications. Technical complications such as root perforations, instrument fractures, or failure to locate root canal orifices have been reported in over 15% of treated maxillary incisors and up to 70% of mandibular incisors. These procedural complications had a significant impact on treatment outcomes. In teeth without complications, healing of pre‐existing apical periodontitis was observed in nearly 90% of cases after 4 years. However, when technical complications occurred during treatment, the success rate dropped noticeably to just 50%.

Another study from the 1980s reported that the success of root canal treatment in teeth with pulp canal calcifications largely depended on the initial periapical status [23]. While teeth with a healthy periapical status showed success rates exceeding 90%, those with calcified canals and pre‐existing apical periodontitis had significantly lower success rates, around 60%. This difference in success rates may likely be due to the difficulty, or in some cases, impossibility of gaining access to the calcified root canal orifice, which is a prerequisite for further successful root canal treatment. In addition, the location of canal calcification has a significant impact on treatment success. Calcifications located in the coronal third can be managed with a success rate of up to 88%, whereas success rates decrease substantially when the calcification is located in the apical third, dropping to 49% [24].

A more recent investigation demonstrated that treatment by an endodontic specialist, supported by optical magnification using an operating microscope and adjunctive tools such as ultrasonic tips, can significantly improve treatment outcomes in cases of pulp canal calcification. In this retrospective study of 41 teeth with a total of 114 calcified root canals in elderly patients managed by an endodontics specialist using state‐of‐the‐art equipment, it was shown that all root canals were accessed successfully, but up to 60 min were sometimes required to locate and negotiate apically displaced and severely narrowed canal orifices due to calcification [25]. Despite high magnification and co‐axial illumination for optimised visualisation of visible structures within the access cavity, extensive removal of dentine may be necessary to provide spatial orientation within the pulp chamber of calcified teeth. This increased removal of tooth structure may compromise the tooth biomechanically and has been associated with a higher risk of fracture [26]. Consequently, even if root canal treatment is technically successful from an endodontic perspective, the long‐term prognosis for tooth retention may still be adversely affected.

## 4 Static Guided Navigation

To enhance precision, efficiency and safety in managing teeth with pulp canal calcification, a computer‐assisted treatment technique, which enables the minimally invasive localization of calcified root canal orifices, was introduced in several publications by different authors in 2015 and 2016 [27, 28, 29, 30]. Since its introduction, research on this technique has expanded globally, with numerous institutions contributing to the growing body of literature. The term ‘Guided Endodontics’ has become widely adopted and is now commonly used as a keyword in international publications [31].

The concept of “Guided Endodontics” is based on principles derived from static computer‐assisted implant surgery, which had already been extensively described and scientifically validated by the time the first publications on Guided Endodontics appeared [32]. Preoperative three‐dimensional digital planning of the endodontic access cavity requires both an intraoral digital surface scan of the dental arch and high‐resolution 3D imaging. Cone‐beam computed tomography (CBCT) is recommended for this purpose, ideally with a limited field of view to ensure the highest possible image resolution through minimal voxel size. In addition to enhanced image quality, a smaller scan volume also reduces radiation exposure for the patient. Although CBCT imaging is associated with a higher radiation dose compared to conventional intraoral radiographs, its use is supported by current guidelines. In its 2019 consensus statement, the European Society of Endodontology recommends considering CBCT imaging when root canal treatment is indicated and severe pulp canal calcification is present, provided that the potential diagnostic and therapeutic benefits outweigh the associated risks of higher radiation doses for patients [33]. In dedicated planning software, a virtual, dimensionally accurate model of the bur intended for clinical use is positioned at the orifice of the apically displaced root canal. This alignment follows the canal's trajectory to achieve straight‐line access, thereby facilitating subsequent canal negotiation with hand files or rotary nickel–titanium instruments. After precise registration of CBCT and intraoral scan data, a template can be digitally designed based on the intraoral scan and subsequently manufactured either subtractively (CAD/CAM milling) or additively (3D printing). A metal sleeve corresponding to the bur is inserted into the template, which is then positioned on the dental arch. This setup enables a statically guided preparation of the access cavity along the preoperatively planned path and angulation.

**FIGURE 1:**
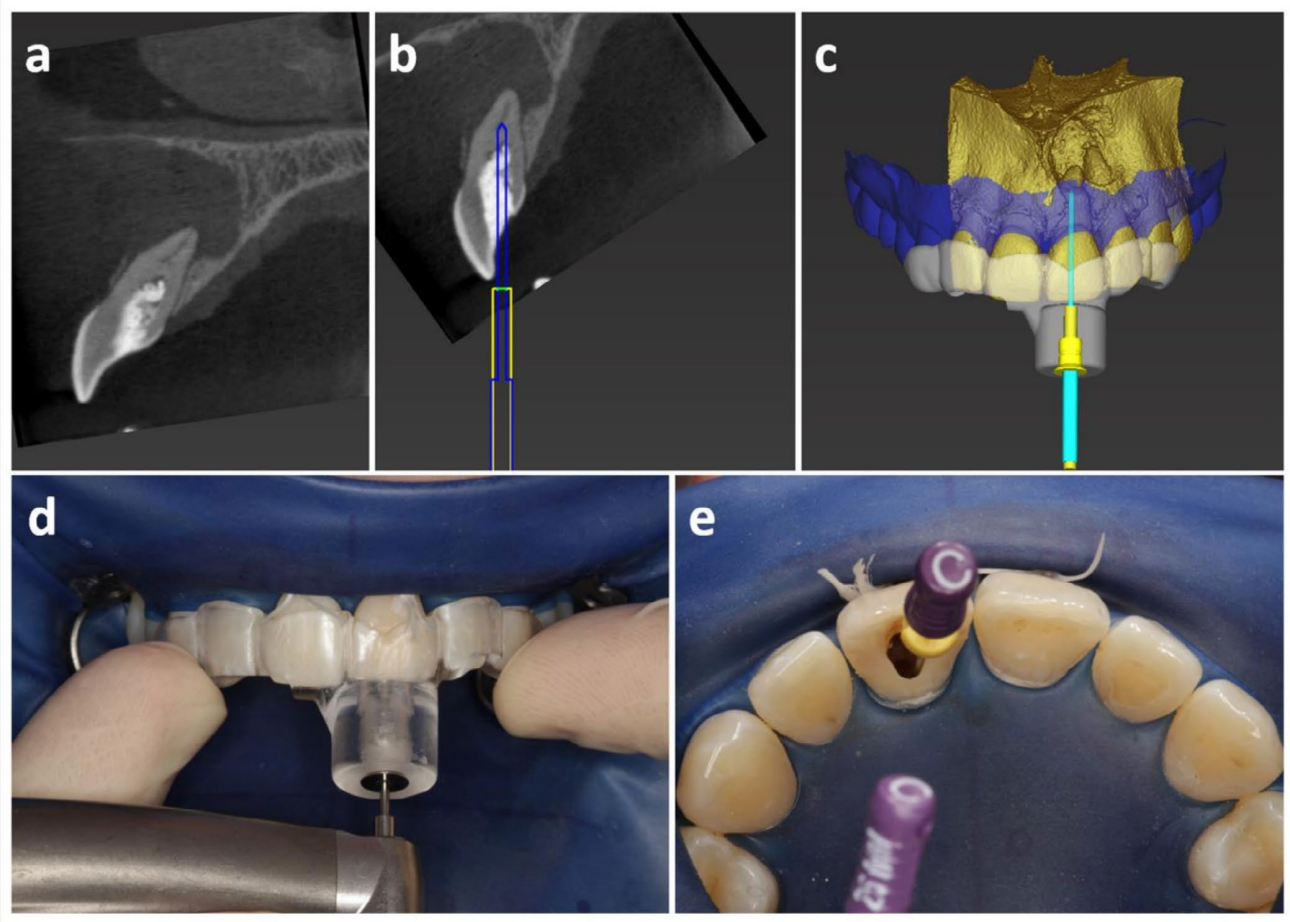
Case presentation of a template‐guided endodontic access cavity preparation (adapted from Connert et al. [34]). (a) Preoperative CBCT showing an unsuccessful root canal localization attempt by the referring dentist in a tooth severely affected by pulp canal calcification. (b) Virtual planning of the access cavity to the orifice of the calcified root canal. (c) Three‐dimensional representation of the virtual planning including the template. (d) Clinical execution of the guided access cavity preparation under rubber dam isolation. (e) Successful localization of the root canal.

The burs used for Guided Endodontics are comparable to conventional carbide burs typically employed for post space preparation and are specifically designed to cut efficiently in dentine. However, enamel at the entry point of the access cavity must be removed beforehand using a conventional diamond bur. The area of enamel requiring removal can be marked by coating the tip of the guided bur with dye (e.g., caries detector or plaque disclosing agent) and inserting it passively through the sleeve until it contacts the enamel surface. The stained enamel region is then selectively and conventionally removed in a non‐guided manner. Care must be taken to create a flat platform onto which the bur can engage as orthogonally as possible. Contact of the bur at an inclined surface may result in deviation of the preparation path, despite static guidance. This is primarily due to the necessary clearance between the bur and the guide sleeve, which is required to avoid friction and excessive heat buildup during preparation. Continuous irrigation and removal of dentine debris maintain cutting efficiency and minimize heat generation. The apical endpoint of the guided access preparation is precisely defined by the stopper of the bur against the metal sleeve, effectively preventing inadvertent overextension toward the apex. Following access cavity preparation using the template, root canal orifice localization can be performed by using a hand file for root canal scouting. Once canal access has been successfully established, chemo‐mechanical preparation proceeds according to conventional protocols involving both manual and rotary instrumentation, along with appropriate endodontic irrigants. Figure 1 shows the treatment of a calcified root canal using template‐guided access cavity preparation.

## 5 Overview of Present Literature

### 5.1 Accuracy and Substance Loss

A growing body of clinical case reports and in vitro studies has highlighted Guided Endodontics as a promising treatment modality and a viable alternative to conventional freehand access cavity preparation. As early as 2016, investigations demonstrated minimal spatial deviation between the digitally planned and the actually prepared access cavities. Angular deviations of less than 2° have been reported, which, depending on the depth of preparation, can result in an apical deviation of less than 0.5 mm [30]. This high level of accuracy is associated with a consistently high success rate in locating calcified root canals.

In addition to its high precision, guided access cavity preparation has been shown to preserve significantly more tooth structure than the conventional freehand method, supporting a more minimally invasive approach—an outcome consistently reported in a 2023 systematic review of the available literature on the topic [35].

Notably, the success of Guided Endodontics appears to be largely independent of the clinician's experience level. Unlike traditional access cavity preparation, which is technique‐sensitive and operator‐dependent, this guided approach can be effectively implemented by general practitioners. It allows for outcomes comparable to those achieved by experienced endodontic specialists using operating microscopes and preoperative CBCT imaging [36].

### 5.2 Modification of Template Designs and Fabrication Procedures

A modification of the previously established Guided Endodontic approach was introduced in 2021 through a clinical case report documenting the successful treatment of a calcified premolar. In this case, a sleeveless guide was digitally designed to control the contra‐angle handpiece head rather than the bur itself, using lateral guiding rails for static navigation. This allowed the clinician to use any compatible bur rather than being limited to a specific one defined during the preoperative planning process. Additionally, this technique offers the advantage of reduced vertical space requirements, addressing one of the main limitations of guided endodontics in cases with limited mouth opening [37].

Another modification of template manufacturing includes the use of an Selective Laser Melting (SLM) approach [38, 39]. Sleeveless, open‐frame titanium templates may offer cost‐related advantages by eliminating the need for specific burs and metal sleeves, while also enhancing water cooling efficiency and thereby reducing heat generation during preparation.

In addition, the development of single‐tooth templates has introduced a more compact design, which may offer advantages over larger conventional templates by facilitating the application of a rubber dam at the onset of the endodontic procedure [40].

A comparative study published in 2020 evaluated the additive (3D printing) and subtractive (CAD/CAM milling) manufacturing techniques for the production of templates. The results indicated that milled (CAD/CAM) templates yielded slightly lower deviations in guided access cavity preparations. Nevertheless, both manufacturing methods enabled successful access cavity preparations and high root canal detection rates [41]. With ongoing advancements in 3D printing technology [42] and the development of improved resins exhibiting reduced shrinkage behaviour, it is anticipated that templates of comparable precision will become achievable. Consequently, the manufacturing method is unlikely to significantly impact the overall accuracy of static Guided Endodontics in the future.

### 5.3 Further Options for Guided Endodontics

Beyond the management of pulp canal calcification, static navigation techniques have also been explored for a range of additional applications, including the treatment of anatomical variations like dens invaginatus [43], retrieval of glass fibre posts [44], access to root canal orifices through hydraulic calcium silicate cements [45], and management of separated instruments [46, 47].

### 5.4 Clinical Studies

To date, two clinical studies have investigated the application of guided endodontics in patient care. An observational study published in 2019 included 50 patients with pulp canal calcification in single‐rooted mandibular and maxillary anterior teeth [48]. The study reported a 100% success rate in canal localization, with significantly higher precision scores observed in mandibular compared to maxillary teeth. Although the procedure time for the guided access preparation was not systematically recorded, the authors estimated a duration of under 2 min. This highlights a substantial time‐saving advantage over the conventional freehand approach, which typically requires considerably more time, even in specialist settings [25].

A more recent clinical study, conducted as a single‐arm prospective, non‐randomised trial with a matched‐pair control group, included a total of 133 teeth [49]. Access cavity preparation for maxillary and mandibular anterior teeth and premolars with calcifications was performed by a single operator in the static guided group (test group), while in the control group, a specialist in endodontics carried out the procedure freehand, supported by preoperative CBCT imaging and an operating microscope. Calcifications in the treated cases were reported to present an average distance of 11–12 mm from the incisal edge or occlusal plane to the canal orifice, as determined by CBCT measurements taken along an axis perpendicular to the tooth. In the test group, successful localization of the root canal orifice was achieved in 59 out of 60 teeth, with no perforations reported. In contrast, the control group using a freehand approach achieved successful access in only 59 out of 73 teeth; seven canals could not be located, and seven perforations occurred. These findings underline the significantly superior clinical outcomes of Guided Endodontics compared to conventional freehand techniques.

## 6 Limitations of Guided Endodontics

One limitation of Guided Endodontics is its reliance on three‐dimensional imaging, typically cone‐beam computed tomography (CBCT), which involves exposure to ionising radiation. An experimental study has demonstrated the potential use of magnetic resonance imaging (MRI) as a radiation‐free alternative for planning guided endodontic procedures [50]. However, dedicated dental MRI systems are still in the early stages of development, and this technique has yet to be applied in a clinical setting. As MRI technology continues to evolve and gain broader adoption in dental and endodontic diagnostics, its integration into Guided Endodontics workflows may become feasible in the future.

For imaging severely calcified root canals, a high‐resolution CBCT scan is generally recommended. However, with currently available CBCT machines, achieving high resolution (small voxel size) typically necessitates the use of a small field of view (FOV). While this enhances image detail, a limited FOV can hinder semi‐automatic three‐point registration with intraoral scans, thereby increasing the risk of registration errors, particularly in the presence of scattering artefacts from radiopaque restorations [51], which may lead to procedural inaccuracies. Such limitations have already been well described in the field of guided implant surgery [52, 53]. The incorporation of radiographic markers during CBCT acquisition can enhance registration accuracy [54], but this requires prior planning by the clinician before image acquisition. Regardless of the method used, all automatic registrations should be carefully reviewed to ensure proper alignment of CBCT and intraoral scan data. In cases of mismatch, most software solutions allow for manual correction. Since inaccurate registration of 3D imaging and intraoral scan data is recognised as a major source of error in guided implant surgery, the same concerns are equally relevant for Guided Endodontics.

The limitation of template‐guided techniques in posterior teeth, particularly premolars and molars, due to restricted vertical space, can potentially be addressed through the use of sleeveless templates [37].

Another limitation of static navigation is its restricted applicability to straight root canals or the straight coronal segment of curved canals. A case report published in 2020 described the treatment of a maxillary canine with severe pulp canal calcification, in which static navigation was performed, reaching a depth of 1 mm distant from the apex [55]. However, due to a slight curvature in the apical third, the root canal could not be negotiated, and apical patency was not achieved. Laser‐activated antimicrobial photodynamic therapy was then applied. Follow‐up at 12 months confirmed periapical healing of a preexisting lesion, suggesting that despite the limitations of static navigation in negotiating canal orifices beyond a curvature, adequate disinfection and infection control were possible without the need for endodontic microsurgery.

The time required for virtual planning and template fabrication poses a challenge for managing patients with pulp canal calcification who present with acute symptomatic apical periodontitis, making immediate intervention in emergency pain situations often not feasible with Guided Endodontics.

## 7 Reported Treatment Failures

Failures associated with Guided Endodontics are rarely reported. Nevertheless, deviations between the planned and the actual access cavity preparation or even perforations can occur, potentially due to planning inaccuracies or an imprecise fit between the guide sleeve and the bur. A case report published in 2022 described the management of such a complication, which ultimately required endodontic microsurgery [56]. The treatment resulted in the successful resolution of the case, with healthy apical conditions documented 2 years following the initial failure of the Guided Endodontics procedure.

## 8 Thermal Damage

As with static computer‐assisted implant surgery, concerns have been raised regarding heat generation during Guided Endodontics, which may pose a risk of thermal damage to cementoblasts and periodontal ligament cells during access cavity preparation. Two experimental studies have investigated this aspect [57, 58]; however, the results were inconsistent, reporting temperature increases ranging from 5°C to 14°C. Moreover, the absence of simulated clinical conditions in these studies limits the ability to extrapolate their findings reliably to clinical practice. Our own, not yet published data show that no overheating of the periodontal ligament is to be expected when the access cavity preparation is performed at 1–2 mm intervals.

## 9 Treatment Costs and Effectiveness

As with many technological innovations in the medical field, Guided Endodontics is associated with increased costs and extended preoperative preparation time. The need for cone‐beam computed tomography (CBCT), intraoral scanning, planning software licenses, template fabrication, and dedicated bur‐sleeve systems contributes to the overall expense of the procedure. Nevertheless, in cases where the technique enables the preservation of teeth that would otherwise require extraction, the treatment may prove to be more cost‐effective than extraction followed by implant‐supported crown restoration. Despite the growing use of cost‐effectiveness analyses in public health research in dental medicine, this aspect of Guided Endodontics has yet to be systematically evaluated.

## 10 Dynamic Navigation

**FIGURE 2:**
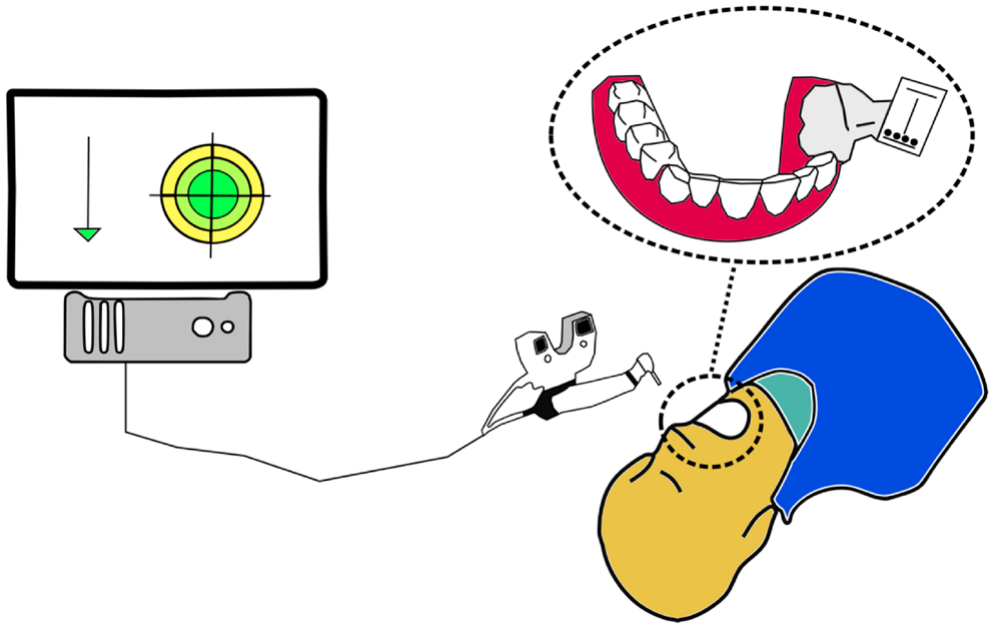
Principle of dynamic navigation. A reference marker is positioned intraorally. A stereoscopic camera system is connected to a computer running dedicated software that enables real‐time navigation. Spatial, angular, and depth deviations are continuously monitored and displayed throughout the procedure (adapted from Connert et al. [34]).

An advancement of static navigation using templates is the application of dynamic navigation systems. Similar to the adoption of the static guided endodontics workflow, this approach builds upon technology previously established in oral implantology. With the introduction of dynamic computer‐assisted implant surgery, it was possible to replace static sleeve‐containing templates with dynamic navigation systems [59]. These systems typically consist of marker‐camera units capable of tracking an instrument (e.g., bur) and displaying its real‐time anatomical position within the patient's mouth to the operator on a screen. Through real‐time visualisation of the spatial position and angulation of a rotating instrument (e.g., diamond bur or carbide round bur) and its deviation from the preoperatively planned ideal position, the operator is dynamically guided to the preoperatively determined endpoint of the access cavity in the CBCT scan—the orifice of the calcified root canal (Figure 2).

Dynamic navigation systems may offer several potential advantages compared to static guidance techniques. These include enhanced visual access to the operative field, increased manoeuvrability in anatomically restricted areas, and the possibility for real‐time intraoperative adjustments to instrument position or angulation. Additionally, the absence of physical templates may facilitate more effective irrigation and thermal control during preparation.

The application of dynamic navigation in endodontics has been reported in the literature as advantageous for both non‐surgical [60, 61] and surgical procedures [62, 63]. Although the primary advantages of dynamic navigation are most evident in anatomically constrained regions such as the posterior dentition, particularly the molar area, the majority of existing studies on its use for access cavity preparation in calcified teeth have focused on anterior teeth and premolars [64, 65, 66, 67], reporting overall benefits in terms of canal detection rates and preservation of tooth structure. In contrast, fewer studies have addressed the use of dynamic navigation in molars [68, 69]; however, the available evidence also supports its efficacy in these cases, demonstrating high rates of canal detection in calcified teeth while maintaining a minimally invasive approach with reduced substance loss compared to conventional freehand techniques.

As with static navigation, it has been suggested that dynamic navigation systems may enable less experienced general practitioners to achieve outcomes comparable to those of endodontic specialists [70]. However, failures involving deviations between the planned and the actual access cavity have also been documented in the literature [71]. In this clinical case series, the root canal orifice could not be successfully located in two out of seven teeth using dynamic navigation alone. Successful treatment was ultimately achieved by an experienced endodontist through the combined use of cone‐beam computed tomography and a dental operating microscope. Moreover, a steep learning curve has been reported with the clinical implementation of dynamic navigation systems [72], with evidence suggesting that the treatment of approximately 18–28 clinical cases may be required to achieve optimal accuracy.

Limitations and disadvantages associated with dynamic navigation systems have been reported in the literature [73]. Currently, only a limited number of systems are commercially available, and the associated technical equipment is highly cost‐intensive, with the most affordable systems starting at approximately $20,000 [73]. Similar to static navigation, dynamic systems require three‐dimensional imaging, which is associated with increased radiation exposure. Although some manufacturers have succeeded in miniaturising camera and marker components [69], many systems still rely on bulky, extraoral markers, which may compromise both patient and operator comfort during clinical procedures to track the position of both patient and instrument in 3D space. Lightweight and ergonomically compact components are advantageous for clinicians, as they may reduce hand and wrist strain [74] during procedures and allow greater flexibility in patient positioning by minimising interference from the navigation hardware.

## 11 Augmented Reality

The integration of augmented reality (AR) represents the next intuitive advancement in endodontic navigation, moving beyond the constraints of current dynamic guidance systems. AR aims to overlay clinically relevant information directly onto the practitioner's field of view, thereby reducing or even eliminating the cognitive burden of correlating data from external monitors to the operative field. However, the clinical implementation of AR in endodontics presents considerable technical challenges, requiring real‐time coordination of multiple components with sub‐millimetre precision to achieve clinical viability.

At the core of AR‐enabled workflows is the continuous acquisition of the clinical scene, typically via optical sensors such as RGB cameras, depth cameras, or structured light systems. These sensors must capture both visual and spatial information with sufficient resolution to meet the high precision demands of endodontic procedures. Moreover, the sensor systems must maintain robustness under variable lighting conditions, occlusions, fluid presence, and movement of the patient or clinician.

Following data acquisition, the system must accurately detect, track, and analyse key objects, including the target tooth, surgical instruments, and anatomical landmarks. A critical step in this process is six degrees of freedom (6‐DoF) pose estimation, which determines each object's spatial orientation and position within the clinical scene to enable accurate alignment of virtual augmentations. In current proof‐of‐concept studies, tracking has been achieved by manual alignment of digital 3D models to in vitro specimens [75] or through point‐based registration using image landmarks [76]. However, these methods can be unreliable in vivo due to variations in landmark visibility, patient movement, and limited or indirect fields of view, particularly when working with dental mirrors. An alternative approach involves the use of extraoral fiducial markers to track both instruments and anatomical models [77], a method that mirrors dynamic navigation systems but requires preoperative calibration and introduces additional setup complexity. Emerging machine learning‐based registration techniques, including deep learning‐driven object pose estimation [78], offer a promising avenue to improve robustness and real‐time accuracy in clinical AR applications.

Equally critical to the clinical utility of AR is the design of intuitive and minimally intrusive visualisations. Basic overlays, such as CBCT‐derived 3D tooth models generated via manual or AI‐based segmentation [79], can aid orientation during access cavity preparation. More advanced visualisation techniques include planned drill trajectories displayed as target lines, which have demonstrated positional accuracy of 0.51 mm at a depth of 4 mm with an angular deviation of 8.5° in in vitro settings [76]. Furthermore, real‐time tracking of both instruments and tooth structures enables visualisation of tool angulation and depth, offering procedural guidance [80]. The incorporation of dynamic visual widgets may further enhance intraoperative feedback [81]. However, while these approaches improve precision, they can also introduce workflow complexity and potential user frustration [81].

Lastly, endodontic specialists often rely on subtle visual cues—such as colour changes in dentine or enamel and spatial orientation within the pulp chamber—to guide their procedures. Advanced computer vision models could be trained to recognise and highlight these features, effectively transferring expert‐level visual insights to general practitioners without necessitating extensive additional training. Such approaches could be further combined with direct visualisation techniques, such as shortwave infrared transillumination, to reveal pulpal structures in real time [82, 83].

Finally, AR content must be delivered through ergonomically suitable interfaces. Currently, the most commonly used devices are external displays and optical see‐through head‐mounted displays [75, 76, 80], though these remain limited in terms of spatial precision. AR‐capable loupes and glasses [84], as well as AR‐integrated surgical microscopes, may offer more practical solutions for dental applications; however, these technologies have yet to be systematically investigated within the field of endodontics.

Although early proof‐of‐concept studies have demonstrated the feasibility of AR‐assisted access cavity preparation and navigation, substantial technical challenges remain, and rigorous clinical validation studies are required before advanced digital navigation technologies can be integrated into routine endodontic practice.

## 12 Concluding Remarks

Guided Endodontics—encompassing static navigation, dynamic navigation, and augmented reality—marks an interesting step toward more precise and minimally invasive root canal treatments. Each approach offers specific advantages depending on clinical needs and available technology. While current evidence supports their efficacy in managing complex cases, further (clinical) research is needed to refine indications and optimize workflows. As digital tools continue to evolve, guided endodontics might become a valuable asset in everyday clinical practice.

## Ethics Statement

The authors have nothing to report.

## Consent

Informed consent was obtained from the patient for the use of clinical and radiological images shown in Figure 1.

## Conflicts of Interest

The authors declare no conflicts of interest.

## Data Availability

The authors have nothing to report.
